# Application of the Technology Acceptance Model to Predict Nursing Students’ Intention to Use Informatics: Cross-Sectional Study

**DOI:** 10.2196/85385

**Published:** 2026-04-13

**Authors:** Habib Alrashedi, Nader Alnomasy, Romeo Jr Mostoles

**Affiliations:** 1 Medical-Surgical Nursing Department, College of Nursing, University of Hail, Baqaa Street, Hail, 55439, Saudi Arabia, 966 0507306911; 2 Mental Health Nursing Department, College of Nursing, University of Hail, Hail, Saudi Arabia

**Keywords:** nursing informatics, technology acceptance model, TAM, digital health, nursing students, informatics

## Abstract

**Background:**

Nursing informatics is essential for digital health transformation; however, the technology acceptance of undergraduate nursing students in Saudi Arabia remains underexplored.

**Objective:**

This study examined factors influencing nursing students’ intention to use informatics technologies using the technology acceptance model.

**Methods:**

A cross-sectional survey was conducted with 132 undergraduate nursing students. Data were analyzed using descriptive, correlational, and hierarchical regression analyses.

**Results:**

Perceived usefulness (mean 3.68, SD 1.22) and perceived ease of use (mean 3.64, SD 1.32) were the strongest predictors of acceptance, together explaining 87% of the variance (*R*²=0.87; *β*=0.323 for usefulness, *P*<.001; *β*=0.195 for ease of use, *P*=.032). Only 25.8% (n=34) of the students often used electronic health records, while 31.8% (n=42) had no electronic health record experience, indicating a clear gap in practical informatics exposure.

**Conclusions:**

Nursing students’ acceptance of informatics is primarily driven by its perceived usefulness and perceived ease of use. These findings highlight the urgent need to integrate practical, user-centered informatics training and clinical simulation into undergraduate nursing curricula to better prepare students for technology-based practice.

## Introduction

The integration of nursing informatics has emerged as a key issue in improving health care delivery, particularly in rapidly changing contexts [1,2], such as Saudi Arabia. Nursing informatics, which serves to bridge information science, computer science, and nursing, can make substantial advances in nursing practice and management through advanced technology usage [3-5]. The effective adoption of systems such as electronic health records (EHRs), clinical decision support tools, and computerized physician order entry is vital for reducing medical errors [6], enhancing patient safety [7], improving care coordination [8], and directly supporting national quality goals. Considering the ambitious objectives of Saudi Arabia’s Vision 2030 to digitize and transform its health care industry [9], the practicality of technologies used by the next generation of practitioners is important and perhaps even imperative. Despite the emerging national need, the acceptance and ability of undergraduate nursing students to engage in nursing informatics for implementation are quite concerning and warrant further research [8,10-12].

Recent studies have highlighted issues related to informatics education in Saudi Arabia. In general, nursing students are aware of the importance of nursing informatics; however, their practical acceptance and use are limited in scope for several related reasons, such as a lack of curricular content, limited clinical experience with informatics, and a lack of consistent faculty support [10,13]. In these attempts, there is a definite disconnect between the theory taught and the actual informatics competencies students need in the clinical environment. This highlights the need for the reform of education due to its limited examples of hands-on training and the development of curricular content that aligns more closely with the current informatics standards in health care delivery [14-17]. Thus, the primary research problem addressed by this study was the lack of empirical understanding of the factors that specifically predict the adoption and use of nursing informatics among undergraduate nursing students in Saudi Arabia, which is a necessary step before effective educational interventions can be designed.

To provide an empirical investigation of this gap, this study uses the established technology acceptance model (TAM) and aims to precisely investigate how undergraduate nursing students’ personal perceptions—namely, perceived usefulness (PU) and perceived ease of use (PEOU) of informatics systems—predict their behavioral intention to use those technologies. This study provides a link between identified gaps in the curriculum, experiences, and measurable psychological factors.

A compelling theoretical framework is required to determine the gaps in this study. TAM, developed by Davis [18], is one of the most important and widely used models for studying users’ willingness to adopt new technologies. This framework helps us study their main perceptions, PU, and PEOU of nursing informatics systems. The current literature helps to report these general barriers, but a quantitative study using the published and validated TAM framework that focuses on and measures acceptance of these general perceptions of nursing informatics by Saudi Arabian nursing students is nonexistent.

This study extends the core TAM by incorporating contextual factors relevant to nursing informatics education. Social influence was conceptualized in line with TAM2 [19], reflecting perceived expectations from peers, faculty, and clinical environments. Engagement and Sustainability were included as contextual constructs, capturing students’ interactions with informatics technologies and their perceptions of their long-term relevance in clinical practice. These variables were examined as supplementary variables rather than primary predictors of acceptance. Although more recent models, such as TAM3 and the unified theory of acceptance and use of technology, incorporate additional contextual and organizational factors, the original TAM was selected for its parsimony and proven applicability in educational and early-stage technology adoption contexts.

As such, this study used the TAM framework in a quantitative study on nursing students’ acceptance of nursing informatics in Saudi Arabia. Using the TAM framework, this study is a cross-sectional survey focused on how PU and PEOU affect students’ behavioral intention to adopt informatics and provides empirical research and direction for educational policy and curriculum development in alignment with Vision 2030. Despite the recognized importance of nursing informatics in supporting digital health transformation, undergraduate nursing students in Saudi Arabia continue to have limited practical exposure to and variable acceptance of informatics technologies. The factors influencing the intention to adopt and use these technologies remain insufficiently understood, particularly within a theory-driven framework.

Therefore, this study aimed to identify factors that predict undergraduate nursing students’ acceptance of nursing informatics technologies using the TAM. The following research questions guided this study: What are undergraduate nursing students’ perceptions of nursing informatics in terms of PU, ease of use, and acceptance? What relationships exist between the core TAM constructs and nursing students’ acceptance of informatics technologies? Which TAM-related factors significantly predict undergraduate nursing students’ acceptance of nursing informatics technologies?

## Methods

### Research Design

This study used a quantitative, cross-sectional survey to investigate the factors affecting the intention to use informatics technologies, using the TAM as a framework. A cross-sectional survey design was selected for its practicality and feasibility in capturing undergraduate nursing students’ perceptions and acceptance of informatics technologies at a single point in time. This design is appropriate for examining the relationships among TAM constructs and identifying key predictors of acceptance without the resource demands associated with longitudinal data collection. This study is reported in accordance with the STROBE (Strengthening the Reporting of Observational Studies in Epidemiology) statement guidelines (Checklist 1) [20].

### Population and Sampling

This study involved third- and fourth-year undergraduate nursing students from the University of Hail in the Hail Region, Saudi Arabia, from October 2025 to December 2025. Participants were recruited using convenience sampling from students who had successfully completed a nursing informatics course and had relevant clinical experience. All qualified students were contacted via a university communication system. This sampling strategy may limit the generalizability of the results; however, it also facilitates the recruitment of students with proper knowledge and clinical experience. Eligible participants were third- and fourth-year undergraduate nursing students who had successfully completed the nursing informatics and core clinical nursing courses, including supervised clinical training as part of the undergraduate curriculum. Completion of these courses ensured that participants had foundational theoretical knowledge of nursing informatics and prior exposure to clinical practice environments in which health information technologies were used.

A priori power analysis was conducted using G*Power software (Mac and Windows, University of Düsseldorf) for hierarchical multiple regression. The analysis assumed an α level of .05, statistical power of 0.80, and a medium effect size (*f*²=0.15). The analysis indicated a minimum sample size of 118 participants. A total of 150 students were invited, of whom 132 completed the survey, yielding a response rate of 88%.

### Instrument

This study is comprised of 2 main parts. The first part explored the demographic and technology profiles of undergraduate nursing students participating in the study. The variables of interest included age, sex, year of study, comfort level with digital technology, previous experience using EHRs, computing access, frequency of technology use for educational purposes, Internet access at home, and planned area of nursing work.

The second part used the TAM questionnaire, modified from Davis [18], to measure students’ perceptions and behavioral intentions regarding their use of health informatics technologies. The instrument measured 3 core constructs: PU, PEOU, and behavioral intention (BI). Items that further explored issues such as subjective norms (SNs; social influence), sustainability, engagement, and willingness to learn new technologies were also assessed. The TAM items were adapted from a study by Davis [18] and expanded to include domains relevant to contemporary digital health education, namely sustainability, engagement, and willingness to learn new technologies. The final instrument consisted of 31 items distributed across 7 constructs: PU (6 items), PEOU (6 items), SNs (2 items), sustainability (4 items), engagement (7 items), and learning new technologies (6 items). Participants rated their responses on a 5-point Likert scale (from 1=“Strongly Disagree” to 5=“Strongly Agree”), and the total scores ranged from 12 to 60. Higher ratings indicated that nursing students had more positive opinions about the usability of artificial intelligence in health care. The questionnaire was administered in English, which is the official language of instruction in the nursing program, and all participants were proficient in English. Therefore, no translation or back-translation procedure was required.

Minor wording adjustments were made solely to contextualize the items to nursing informatics and EHR use (eg, replacing generic references to “technology” with “nursing informatics systems”), without changing the meaning or theoretical intent of any item. The underlying structure and theoretical integrity of the original TAM instrument were maintained.

Prior to the main data collection, the instrument was pilot-tested with 20 second-year undergraduate nursing students to assess its clarity, reliability, and internal consistency. These students were excluded from the final sample. Feedback from the pilot test resulted in minor wording refinements to improve item clarity, while the overall structure of the instrument remained unchanged. Internal consistency demonstrated good-to-excellent reliability across constructs in the pilot test, with Cronbach α values of PU=0.953, PE=0.900, SN=0.929, sustainability=0.917, engagement=0.932, and learning new technologies=0.911, indicating satisfactory psychometric properties of the instrument.

### Ethical Considerations

After obtaining institutional review board approval from the University of Hail (H-2024‐437 on September 16, 2024), an ethical recruitment method was established. The purpose of the study was shared with the nursing students in the classroom, and the students were invited to participate. A link to the survey tool was distributed to the students via WhatsApp numbers and university email addresses. Data were collected electronically using Google Forms. Informed consent was obtained electronically through Google Forms. The anonymity and confidentiality of the participants were maintained throughout the study. Moreover, participants were informed that there were no incentives or compensations for participating in this study. Google Forms was chosen for its ease of access and reliable data storage capabilities, providing an efficient process for collecting information on undergraduate nursing students’ informatics competencies.

The survey began with an introductory page outlining the purpose of the study, assurances of anonymity, and details of voluntary participation. Participation was voluntary, and the students were informed that they could withdraw at any time without penalty or impact on their academic standing. Data were collected anonymously using Google Forms, and no identifying information was obtained. Access to the data was restricted to authorized members of the research team to ensure confidentiality and data security. The study involved minimal risk and adhered to ethical principles throughout the recruitment and data-collection processes.

Two reminders (1 email and 1 in-class announcement) were used to increase the response rates.

### Statistical Analysis

The data were analyzed using IBM SPSS Statistics (version 27). Descriptive statistics were used to summarize participants’ demographic characteristics and study variables. Pearson correlation coefficients were calculated to examine the relationships among the TAM constructs. Hierarchical multiple regression analysis was conducted to identify the predictors of nursing students’ acceptance of informatics technologies. In model 1, demographic and technology-related control variables (age, gender, year level, prior informatics training, exposure to EHRs, comfort with technology, frequency of technology use, internet accessibility, and intended future nursing career) were entered. In model 2, the core TAM variables—PU and PEOU—were added. Model 3 included extended TAM-related variables (SN, sustainability, engagement, and willingness to learn new technologies).

Before the analysis, the assumptions of multiple regression were examined. The normality of residuals and linearity were assessed through visual inspection of the residual plots. Multicollinearity was evaluated using variance inflation factor values, all of which were within acceptable limits. Statistical significance was set at *P*<.05 for all analyses.

## Results

Most participants were female (n=113, 85.6%) and aged 21 to 25 years (n=96, 72.7%). Although access to technology was high, 76.5% (n=101) reported access to a personal computer, and 79.5% (n=105) used technology daily for educational purposes. Comfort with digital technology varied, and clinical informatics exposure remained limited. Only 25.8% (n=34) of the students reported frequent use of EHRs, while 31.8% (n=42) had no prior EHR experience. Clinical care was the most common career pathway (n=80, 60.6%; Table 1).

**Table 1.** Demographic and technological profile (N=132).

Category and subcategory	Value, n (%)
Age (y)	
≤20	31 (23.5)
21-25	96 (72.7)
≥26	5 (3.8)
Sex	
Male	19 (14.4)
Female	113 (85.6)
Year level	
Third year	55 (41.7)
Fourth year	77 (58.3)
Rate your comfort level with digital technology (eg, computers, smartphones, and EHRs^a^)	
Very uncomfortable	18 (13.6)
Uncomfortable	33 (25)
Neutral	36 (27.3)
Comfortable	45 (34.1)
Previous exposure to EHRs	
No experience	42 (31.8)
Yes, occasionally (monthly or less)	56 (42.4)
Yes, frequently (weekly or daily)	34 (25.8)
Do you own or regularly have access to a personal computer or laptop?	
No	31 (23.5)
Yes	101 (76.5)
How often do you use technology (smartphones, tablets, and computers) for educational purposes?	
Monthly	8 (6.1)
Weekly	19 (14.4)
Daily	105 (79.5)
Internet accessibility at home	
Fair	7 (5.3)
Good	36 (27.3)
Excellent	89 (67.4)
Intended future nursing career area	
Undecided	19 (14.4)
Administration or leadership role	7 (5.3)
Academic or teaching role	11 (8.3)
Nursing informatics	8 (6.1)
Public health or community nursing	7 (5.3)
Clinical care (hospital or clinical setting)	80 (60.6)

a EHR: electronic health record.

The descriptive statistics for the study variables are presented in Table 2. Among the TAM constructs, PU (mean 3.68, SD 1.22) and PEOU (mean 3.64, SD 1.32) had the highest mean scores, indicating generally positive perceptions of nursing informatics. Learning new technologies (mean 3.61, SD 1.13) and overall acceptance (mean 3.60, SD 1.24) were also rated positively. In contrast, SN had the lowest mean score (3.21, SD 1.22), suggesting that social influence played a less prominent role in students’ perceptions than individual cognitive factors.

**Table 2.** Descriptive statistics of study variables.

Variable	Value, mean (SD)	
Acceptance	3.60 (1.24)	
Perceived usefulness	3.68 (1.22)	
Perceived ease of use	3.64 (1.32)	
SN^a^	3.21 (1.22)	
Sustainability	3.50 (1.20)	
Engagement	3.35 (1.15)	
Learning new technologies	3.61 (1.13)	

a SN: subjective norm.

As illustrated in Table 3, the acceptance of nursing informatics demonstrated strong positive correlations with PU (*r*=0.83; *P*<.01), PEOU (*r*=0.81; *P*<.01), and learning new technologies (*r*=0.83; *P*<.01). Additionally, PU and PEOU were highly interrelated (*r*=0.82; *P*<.01), supporting the theoretical assumptions of TAM. SNs showed weaker, though still significant, correlations with acceptance (*r*=0.45; *P*<.01), reinforcing the comparatively limited role of social influence in this sample. Despite these strong correlations, multicollinearity diagnostics remained within acceptable limits (variance inflation factor<6), suggesting that the predictors could be reliably included in the regression models.

**Table 3.** Intercorrelations between study variables.

Variable	Acceptance	PU^a^	PEOU^b^	SN^c^	Sustainability	Engagement	LN^d^
Acceptance	—^e^	.83	.81	.45	.79	.69	.83
PU	.83^f^	—	.82	.43	.75	.60	.78
PEOU	.81^f^	.82^f^	—	.43	.77	.61	.78
SN	.45^f^	.43^f^	.43^f^	—	.50	.56	.47
Sustainability	.79^f^	.75^f^	.77^f^	.50^f^	—	.77	.84
Engagement	.69^f^	.60^f^	.61^f^	.56^f^	.77^f^	—	.77
Learning new technologies	.83^f^	.78^f^	.78^f^	.47^f^	.84^f^	.77^f^	—

a PU: perceived usefulness.

b PEOU: perceived ease of use.

c SN: subjective norm.

d LN: learning new technologies.

e Not applicable.

f *P*<.01 (2-tailed).

The hierarchical regression for predicting the acceptance of nursing informatics (Table 4) revealed that, in model 1, only comfort with technology was a significant predictor with a negative effect (β=−0.363; *P*=.05). Model 2 included PU and PEOU, and both variables were significant positive predictors (*β*=0.379; *P*<.001). The final model indicated that PU (*β*=0.323; *P*<.001), PEOU (*β*=0.195; *P*=.03), and learning new technologies (*β*=0.260; *P*=.008) were significant positive predictors.

**Table 4.** Hierarchical regression predicting acceptance with 95% CIs.

Predictor	B	SE	β	*t* test (*df*)	*P* value	95% CI	VIF^a^
Model 1							
Age (y)	−0.136	0.217	−0.053	−0.626 (122)	.53	−0.566 to 0.294	1.11
Gender	−0.080	0.295	−0.023	−0.270 (122)	.79	−0.663 to 0.504	1.07
Informatics training	−0.019	0.213	−0.008	−0.089 (122)	.93	−0.441 to 0.403	1.13
Exposure to EHRs^b^	0.199	0.135	0.122	1.472 (122)	.14	−0.069 to 0.467	1.05
Year level	0.387	0.219	0.154	1.766 (122)	.08	−0.047 to 0.820	1.16
Comfort with technology	−0.427	0.099	−0.363	−4.324 (122)	<.001^c^	−0.623 to −0.232	1.08
Often using technology	−0.250	0.183	−0.113	−1.366 (122)	.18	−0.611 to 0.110	1.13
Internet accessibility	0.145	0.208	0.065	0.695 (122)	.49	−0.267 to 0.557	1.18
Future nursing career	0.032	0.087	0.026	0.365 (122)	.72	−0.140 to 0.203	1.07
Model 2							
PU^d^	0.357	0.077	0.379	4.632 (120)	<.001^c^	0.205 to 0.510	3.16
PEOU^e^	0.347	0.077	0.379	4.632 (120)	<.001^c^	0.205 to 0.510	3.16
Model 3							
PU	0.330	0.083	0.323	3.980 (116)	<.001^c^	0.166 to 0.494	3.94
PEOU	0.207	0.084	0.195	2.481 (116)	.03^f^	0.018 to 0.395	3.99
SN^g^	−0.006	0.054	−0.007	−0.131 (116)	.89	−0.113 to 0.101	1.61
Sustainability	0.092	0.071	0.113	1.294 (116)	.20	−0.048 to 0.233	4.12
Engagement	0.192	0.085	0.185	1.094 (116)	.28	−0.155 to 0.276	3.62
Learning new technologies	0.286	0.105	0.260	2.721 (116)	.008^c^	0.078 to 0.493	5.46

a VIF: variance inflation factor.

b EHR: electronic health record.

c **P<.01.

d PU: perceived usefulness.

e PEOU: perceived ease of use.

f *P<.05.

g SN: subjective norm.

Table 5 summarizes the proportion of variance in the acceptance of nursing informatics explained by predictors across each step of the hierarchical regression model. In model 1, the demographic and technology-related control variables explained 45% of the variance (*R*²=0.45), with an adjusted *R*² of 0.20. In model 2, when PU and PEOU were entered, the model explained 87% of the variance (*R*²=0.87), with an adjusted *R*² of 0.75. In model 3, when SN, sustainability, engagement, and learning new technologies were added, the final model explained 90% of the variance (*R*²=0.90), with an adjusted *R*² of 0.80. The increase in *R*² from model 1 (0.45) to model 2 (0.87) is striking; therefore, PU and PEOU are the primary predictors, and the final model has a very good overall fit.

**Table 5.** Hierarchical multiple regression.

Model	*R* ^2^	Adjusted *R*^2^
1	0.45	0.20
2	0.87	0.75
3	0.90	0.80

Table 6 displays hierarchical regression, revealing that all models predicting acceptance of nursing informatics were statistically significant. In model 1, which included demographic and technological controls, the regression was significant (*F*_9,122_=3.503; *P*<.001), accounting for a small amount of variance in acceptance. Model 2, which included the core TAM constructs (PU and PEOU), showed a substantial increase in the variance explained (*F*_11,120_=32.442; *P*<.001), confirming the primary role of these 2 constructs in acceptance behavior. Model 3 also showed a strong statistically significant outcome (*F*_15,116_=32.024; *P*<.001), as it included all 7 study variables and accounted for the largest amount of variance explained. The increasing values of the *F* test and the consistent statistical significance across the models indicate that the contextual variables and TAM constructs work in succession to predict students’ acceptance of nursing informatics.

**Table 6.** ANOVA summary for hierarchical regression predicting acceptance^a^.

Model	Sum of squares (regression)	Sum of squares (residual)	Mean square (regression)	Mean square (residual)	*F *test (*df*)	*P* value
1	41.527	160.702	4.614	1.317	3.503 (9, 122)	<.001^b^
2	151.339	50.890	13.758	0.424	32.442 (11, 120)	<.001^b^
3	162.893	39.336	10.860	0.339	32.024 (15, 116)	<.001^b^

a Analyses were performed using IBM SPSS Statistics (version 27; IBM Corp).

b *P*<.01.

## Discussion

### Perceptions and Acceptance of Nursing Informatics

Nursing students have a strong, positive perception of nursing informatics, recognizing these technological tools as practical resources that contribute to the quality and safety of patient care, which supports national modernization strategies for digital health in the sector. Consistent with the present findings, Al-Olaimat et al [21] reported that perceived usefulness is a strong predictor of nursing students’ acceptance of health information technologies, underscoring the importance of clinical relevance. Similarly, Alnajjar et al [22] found that perceived ease of use significantly influenced technology adoption among nursing students, aligning with our results that usability remained central to informatics acceptance.

However, positive perspectives on nursing informatics and the use of technology are not universal and have exposed serious challenges to implementation. Studies have shown negative or mixed perspectives on nursing informatics and technology use, especially among students who have received little or no training. Given this gap between positive and negative experiences, nursing curricula should incorporate core informatics competencies across all training levels.

### Correlation Between TAM Domains

The high positive correlations found between factors influencing nursing students’ acceptance of informatics technologies and their perceptions of usefulness, ease of use, and learning opportunities indicate that these personal factors are the primary drivers of adoption. This is not surprising, as students are inherently drawn to technologies that optimize their work, are easy to navigate, and directly advance the acquisition of important professional skills; these direct personal advantages ultimately fulfill their pragmatic and professional needs.

Conversely, the study results indicate that external social norms or pressures have significantly less influence. In other words, students rely on their own attitudes and experiences rather than peer or institutional pressure when making adoption decisions.

This finding is consistent with other studies showing that personal direct advantages are significant predictors of technology acceptance by nursing students and clinicians. In a 2025 cross-sectional study by Jallad et al [23], which used TAM as a research framework, students’ personal factors (usefulness, ease of use, human, and technology) were highly positively correlated with informatics adoption. Moreover, their findings suggest that personal advantages still matter even in the presence of institutional support. Similarly, Aldosari et al [24] found a highly positive correlation between PU and PEOU, which led to positive acceptance of health information technologies by nurses. Nguyen et al [25] likewise found that, especially for clinicians, PU and PEOU had an exceptionally influential effect on information and communication technologies acceptance, typically outweighing social influence.

Although these data likely emphasize personal factors, other studies indicate the opposite premise, where external social influence is stronger, at least comparable to, or even better than personal benefits, indicating that context affects the degree of influence. For example, Warshawski [26] found that social influence, performance expectancy, and facilitating conditions were important variables that positively contributed to nursing students’ use of technology in Israel. Furthermore, Garavand et al’s [27] systematic review made similar assertions regarding the importance of social impacts (eg, peer and institutional support), noting that they were not always weighted as heavily as personal factors.

### Factors Influencing the Acceptance of Nursing Informatics

This study explored the factors influencing the prospective acceptance of nursing informatics by Saudi undergraduate nursing students. Overall, the findings highlight individuals’ central and predominant personal perceptions as predictors of technology acceptance in this population, which is congruent with the underlying theoretical principles of the TAM. The input of cognitive and social variables provides institutions with practical recommendations to support the integration of informatics into the nursing education curriculum, which is pertinent, as the Saudi health care system continues to progress through digital changes.

The final structural model provided solid empirical support for the fundamental TAM constructs, highlighting that PU, PEOU, and students’ ability and willingness to learn new skills are statistically significant positive predictors of perceived technology acceptance. The findings demonstrated that PU and PEOU were related to a positive and significant degree of perceived acceptance and were consistent with the fundamental TAM assumptions and an extensive array of empirical literature in health care. PU and PEOU were strongly and positively correlated with perceived acceptance. This is consistent with the fundamental premise of TAM, which posits that students who perceive informatics systems to be useful in their education and future desired clinical experience (PU) and easy to understand (PEOU) will act to adopt the technology. The existing literature, including studies on Saudi nursing students and practicing nurses [24,28-30], suggests that of all the elements explored, only 2 personal cognitive constructs, PEOU and PU, are consistently shown to have the greatest significance. The literature also describes how PEOU and PU are often interrelated, thereby bolstering their interconnected predictive ability [31], which may have contributed to the predictive ability of the present model.

In contrast, the endogenous variables of SN, sustainability, and engagement were not statistically significant, suggesting that these were even less important than personal experience with PEOU and PU. This suggests that social influence (SN), long-term environmental sustainability, and active engagement with technology (sustainability and engagement) did not influence the initiation of acceptance in this student population. While SN being nonsignificant is consistent with some uses of TAM, where personal interest overcomes the social element, sustainability and engagement are not significant and inconsistent with an emerging corpus of published research in the e-learning and health informatics space that has identified that user engagement, perceived enjoyment, and organizational support (variables conceptually associated with sustainability and engagement) could become predictive, particularly if the use was measured in a temporally posterior fashion [32,33].

The initial observation that PU and PEOU are key drivers of technology acceptance, whereas SN, sustainability, and engagement are not statistically significant, has important context. This finding indicates that, for initial adoption, personal internal motivation (use and ease) is more important than social pressure. Similarly, SN, sustainability, and engagement not being significant were probably affected by students’ exposure to informatics systems (eg, EHRs) not being large-scale, and dimensions regarding long-term and organizational support do not have enough influence on study acceptance at the beginning of use.

This is consistent with TAM in the health care sector. Reviews have always supported PU and PEOU as dominant predictors and found that SN and its related constructs were nonsignificant [31]. Health care studies have shown that internal cognitive processes outweigh social influences on clinicians and students in novice settings [24,25,29]. The lack of predictive ability for sustainability and engagement constructs also aligns with the results in the context of a lack of practical experience [34,35].

However, this inverse result was also demonstrated. Research in learning technology or mobile health usually reports engagement or sustainability properties (such as enjoyment or organizational support) as significant [32,33] when examined in a technology-rich or long-term context.

Thus, the differential results highlight an important caveat. Although PU and PEOU drive acceptance for this undergraduate sample, the lack of importance of the other constructs may be a contextual (temporal and situational) feature of the participants’ inexperience when using the systems in their educational (and possibly first) experiences with such systems. Consequently, participants may regard SN, engagement, and sustainability as collectively gaining importance as predictors later (ie, when adults have graduated and worked for a while in actual practice with longitudinal interactions with complex systems). Organizational support and peer influence have a greater impact in an institutional environment and lay the groundwork for future longitudinal studies.

The nonsignificant effects of social influence, engagement, and sustainability may reflect students’ limited and largely theoretical exposure to informatics technologies. At this early stage, acceptance appears to be driven primarily by PU and PEOU, whereas contextual and organizational factors may become more influential during prolonged or mandatory system use in clinical practice. Overall, our findings support the classic TAM perspective that usability and use dominate early acceptance decisions, whereas social factors may play a greater role in long-term use or in highly structured environments.

### Incremental Value of TAM Predictors

The hierarchical regression analysis demonstrated the incremental value of TAM predictors in explaining nursing students’ acceptance of informatics technologies. While demographic and background variables accounted for a moderate proportion of the variance, their explanatory power was substantially enhanced by the inclusion of PU and PEOU, which emerged as the dominant predictors. This finding underscores that acceptance among undergraduate nursing students is driven primarily by individual cognitive evaluations rather than contextual or social factors.

The role of demographic characteristics, ecosystem characteristics, and other common predictors of adoption, such as social influence, is negligible in comparison, further lending credence to the notion that a strategy focused on usability and functionality will best advance informatics acceptance. This pattern is expected, as a substantial amount of evidence has emerged that supports the TAM as the reigning model in health care, consistently identifying usability and PEOU as the primary predictors for nurses’ and other health professionals’ adoption of health records and myriad other systems [24,36,37].

While the first 2 key TAM predictors are statistically reliable, the literature is not homogeneous. However, caution should be exercised when interpreting the extent to which the other variables of technology acceptance are equally or more robust. Some studies, especially those that have combined the unified theory of acceptance and use of technology model or the information system success model as a foundation, offer examples where social influence, engagement, organizational factors, and education are the strongest predictors of technology use, such as in mobile learning [38]. Moreover, reviews have reported that facilitating conditions, technostress, system quality, and satisfaction could be as impactful as, or more impactful than the primary TAM variables, especially in social, mandated, or organizational pressure contexts [35,38,39]. This suggests that PEOU may not always be a direct predictor, as its impact may be mediated by organizational culture, trust, or policy. Additionally, the direct impacts of TAM variables can be diminished or eliminated during periods of strong compliance or external pressure [35,39]. Future research should explore additional factors that may shape nursing informatics acceptance, including technostress, system quality, and organizational support, particularly in clinical settings where informatics systems are mandatory. Longitudinal and multi-institutional studies may help clarify how these variables interact with core TAM constructs over time and across different stages of professional development.

### Limitations of the Study

This study had several limitations. First, the cross-sectional design limits causal inference; longitudinal studies are needed to examine changes in informatics acceptance over time. Second, the data were collected from a single nursing program within one university in Saudi Arabia, which may limit the generalizability of the findings to other institutions or countries. Third, the use of self-reported data may introduce response and social desirability biases, and potential nonresponse bias cannot be ruled out. Finally, participants’ limited prior exposure to EHRs may have influenced their perceptions of informatics acceptance.

### Implication

The results indicate that PU and PEOU are key predictors of the initial acceptance of nursing informatics, reflecting students’ early cognitive evaluations rather than experiential judgment. Given the limited practical exposure to informatics systems among the participants, factors related to sustained use, such as social influence, engagement, and sustainability, were not significant. These findings suggest that while usability and perceived value drive early acceptance, continued use is more likely to depend on prolonged clinical exposure, institutional support, and repeated system interaction, underscoring the need for longitudinal practice-based informatics training in nursing education. Informatics competencies should be explicitly embedded into course learning outcomes to align education with digital health practice requirements. In addition, faculty development programs are needed to prepare educators as informatics “super users” who can effectively support students in technology-rich learning environments. Beyond education, the results are relevant to policymakers and hospital administrators who play a critical role in supporting digital transformation. Investment in standardized training platforms, collaboration between academic institutions and health care organizations, and the provision of supportive clinical informatics infrastructure can facilitate smoother transitions of nursing graduates into digitally enabled health care systems.

### Conclusion

This study demonstrates that undergraduate nursing students’ acceptance of nursing informatics is primarily driven by perceptions of its usefulness and ease of use, despite generally positive attitudes and high access to technology. These findings highlight a persistent gap between theoretical exposure and practical informatics experience, particularly in relation to EHR use. By prioritizing user-friendly, clinically relevant informatics education and simulation, nursing programs can prepare graduates to participate effectively in Saudi Arabia’s ongoing digital health transformation.

## Supplementary material

10.2196/85385 Checklist 1 STROBE checklist.

## Abbreviations

EHR: electronic health record
PEOU: perceived ease of use
PU: perceived usefulness
SN: subjective norm
STROBE: Strengthening the Reporting of Observational Studies in Epidemiology
TAM: technology acceptance model
